# Neonatal septicemia at intensive care unit, Ayder Comprehensive Specialized Hospital, Tigray, North Ethiopia: Bacteriological profile, drug susceptibility pattern, and associated factors

**DOI:** 10.1371/journal.pone.0235391

**Published:** 2020-06-30

**Authors:** Yemane Weldu, Mulugeta Naizgi, Amanuel Hadgu, Abraham Aregay Desta, Amlsha Kahsay, Letemichael Negash, Genet Gebrehiwet Hailu, Araya Gebreyesus Wasihun

**Affiliations:** 1 Department of Medical Microbiology and Immunology, Division of Biomedical Sciences, College of Health Sciences, Mekelle University, Mekelle, Tigray Region, Ethiopia; 2 Department of Paediatric & Child Health, School of Medicine, College of Health Sciences, Mekelle University, Mekelle, Tigray Region, Ethiopia; 3 Department of Public Health Research, Public Health Research and Emergency Management Directorate, Tigray Health Research Institute, Mekelle, Tigray Region, Ethiopia; Panstwowy Instytut Weterynaryjny - Panstwowy Instytut Badawczy w Pulawach, POLAND

## Abstract

**Background:**

Neonatal septicemia is a life threatening medical emergency that requires timely detection of pathogens with urgent rational antibiotics therapy.

**Methods:**

A cross-sectional study was conducted between March 2017 to September 2018 among 317 septicemia suspected neonates at neonatal intensive care unit, Ayder Comprehensive Specialized Hospital, Mekelle, Tigray, North Ethiopia. A 3 mL of blood was collected from each participant. Identification of bacterial species was done using the standard microbiological techniques. Antibiotic sensitivity test was done using disk diffusion method. Data were entered and analyzed using computer software SPSS version 22. Bivariate and multivariate regression analysis was applied to determine the association between variables.

**Results:**

Of the 317 (190 male and 127 female) neonates, 116 (36.6%) were found to be with culture proven septicemia. *Klebsiella* species were the predominant etiologic agents. Length of hospital stay (AOR (adjusted odds ratio) = 3.65 (2.17–6.13), p < 0.001) and low birth weight (AOR = 1.64 (1.13–2.78), p = 0.04) were the factors associated with neonatalsepticemia. Most isolates showeda frightening drug resistance rate to the commonly used antimicrobial drugs. *K*. *pneumoniae*, *E*. *coli*, *Enterobacter* and *Citrobacter* species were 57% to100% resistant to ceftazidime, ceftriaxone, gentamycin, amoxacillin-clavulunic acid and ampicillin. All, 9 (100%) isolates of *S*. *aureus* were resistant to oxacilline, ampicillin,erythromycin and gentamycin. Furthermore, 55.6% *S*. *aureus* isolates were Methicillin Resistant *Staphylococcus aureus*.

**Conclusion:**

Neonaltal septicemia is found to be significantly high in the present study. As most of the isolates are potentially related to hospital acquired infections, prevention and control policy should have to be more strengthening in the neonatal intensive care unit.

## Background

Globally, sepsis is still one of the major causes of morbidity and mortality in neonates [1]. Though tremendous progress in child survival has been made over the past two decades, mortality in neonates remains disproportionately high. Globally, of the deaths that occur in the first five years, about half of them occur within the first 28 days of birth [2], and more than 90% of them are from developing countries [3]. Neonatal death occurring in developing countries is largely from preventable infectious causes like sepsis [4, 5]. In Ethiopia, approximately 42% of the under-5 mortality is attributable to neonatal deaths [6], and sepsis is among the common causes of neonatal mortality [7].

Diagnosis and management of sepsis are a great challenge facing neonatologists in neonatal intensive care units. Although the WHO (World Health Organization) has made criteria for initial diagnosis, clinical diagnosis of neonatal sepsis is difficult due to nonspecific signs and symptoms [8, 9]. The gold standard for diagnosis of sepsis is isolation of the bacterial agent from a blood culture [10].

The spectrum of organisms that cause neonatal sepsis changes overtime and from region to region [11–13]. Nowadays, asdrug resistance is rapidly emerging worldwide; sepsis is continuing to be a potentially disastrous problemdue to fewer treatment options. Alarming degree of drug resistance rate is reported from elsewhere in the globeto the antibiotics like ampicillin, gentamicin, ceftriaxone, ceftazidime, cefotaxime and tetracycline [12–18].

In Ethiopia, since the epidemiology of neonatal sepsis has not been extensively studied, to date most empirical treatments are given based on data from developed countries. Therefore, there is a need for routine bacterial surveillance and study of their susceptibility patterns. Furthermore, it must be also an essential component of neonatal care. Hence, this study was aimed to determine neonatal sepsis; bacteriological profile, drug susceptibility pattern and associated factors at Neonatal Intensive Care Unit, Ayder Comprehensive Specialized Hospital, Tigray, north Ethiopia.

## Materials and methods

### Study area

The study was conducted at Neonatal Intensive Care Unit (NICU), Ayder Comprehensive Specialized Hospital, Mekelle, Tigray region, North Ethiopia. Mekelle is the capital city of Tigray Regional State with a total population of 215,546 (According to the Ethiopian Census data Population statistics projections on 2009). Ayder Comprehensive Specialized Hospital is the largest hospital in Tigray region and the second largest hospital in the nation, which has about 500 beds. The hospital receives referred patients from all parts of the Region (which comprises about 6 million people) and other neighborhood regions such as Afar and Amhara. It also provides local emergency service.

### Study design and study period

A cross-sectional study was conducted from March 2017– September 2018.

### Sample size and sampling technique

Sample size was calculated based on a single population proportion formula as follows:

$$Sample\;Size\;(n)=\frac{z^{2}p\;(1‑p)}{d^{2}}=\frac{{\left(1.96\right)}^{2}*0.321*\left(1‑0.321\right)}{{\left(0.05\right)}^{2}}=335$$

Where; p = prevalence of bacterial isolates(32.1%) which was taken fromprevious report in Gondar, Ethiopia [12], d = degree of accuracy desired (0.05), Z^2^1- α/2 = the standard normal deviation (1.96).

Finally, a total of 317 (94.6% response rate) participants were recruited using aconsecutive convenient sampling technique.

### Requirement criteria

#### Inclusion criteria

After suspicion of neonatal sepsis is made by the attending pediatrician, babies with a clinical diagnosis of neonatal sepsis at the time of admission or during their hospital stay were enrolled in the study.

#### Exclusion criteria

Babies who had received antibiotics before admission or who had criticalcongenital anomalies were excluded from the study.

### Data collection

A structured data collection format derived from the guidelines laid down by WHO young Infant Study Group [19] was used to obtain socio-demographic data and other relevant information such as Intrapartum fever, premature rupture of membranes (PROM), weight of the baby, gestational age, mode of delivery, presence or absence of lethargy, fever, failure to suck, moro reflex, tachypnea, apnea, tachycardia, respiratory distress, seizures, asphyxia and jaundice.

### Laboratory procedures

About 3 ml of whole blood was collectedaseptically and inoculated into Brain Heart Infusion broth (BHI) in a ratio of blood: BHI of 1:10 and transported to the Microbiology laboratory for further processing. After 24 hrs incubation in Brain Heart Infusion broth, sub-culture was done onto blood agar, chocolate agar and MacConkey agar. Broth cultures with negative results in the first 24 hrs incubation were further re-incubated and then sub cultured after 48 hours, 96 hrs, with last sub-culture on day 7. Identification of bacterial colony was made bycolony morphology, gram staining reactions and biochemical reactions such as catalase, coagulase, hemolytic activity, triple sugar iron, indole, motility, citrate, urease and hydrogen sulphide production tests.

Antimicrobial susceptibility pattern of the isolates was determined byKirby disk diffusion method. For gram positive bacteria the following discs were included: gentamicin (10μg),tetracycline (30μg), ciprofloxacin (5μg), clindamycin (2μg), erythromycin (15μg), vancomycin (30μg), oxacillin (5μg), chloroamphinicole (30μg), ceftriaxone (5μg), ceftazidime (10μg), sulphamethaxazole/trimethoprim (25μg), amoxacillin-clavulunic acid (20/10μg), ampicillin (10μg) and cefoxitin (30μg). For gram negatives: gentamicin (10μg), ampicillin (10μg), amoxacillin-clavulunic acid (20/10μg), ciprofloxacin (5μg), tetracycline (30μg), sulphamethaxazole/trimethoprim (25μg), norfloxacilin (10μg), chloroamphinicole (30μg), ceftriaxone (30μg), ceftazidime (10μg), cefotaxime (5μg), amikacin(10μg) and meropenem (10μg) were used.

### Data quality control

Laboratory analyses were carried out using standard operating procedures (SOPs). Prior to the actual work, reagents were checked for proper functioning and expiry dates. All culture media were prepared following the manufacturer’s instructions. Each batch of the prepared media was checked for sterility by incubating a sample medium (5%) at 37^∘^C for 24hrs. Known bacterial species were inoculated and incubated at 37^∘^C for 24hr for the performance check. *Escherichia coli* ATTC 25922, *Staphylococcus aureus* ATTC 25923 and *Pseudomonas aeruginosa* ATTC 27853were used as control strains.

### Data analysis

Data were analyzed using computer software (SPSS version 22). Frequency and percentage were employed to summarize the results and presented in tables. In univariate analysis, all variables witha p-value of < 0.05 were subjected to multivariate analysis. In multivariate regression analysis, to determine the association between variables, a p-value < 0.05 with a corresponding 95% confidence interval was considered as statistically significant.

### Ethical considerations

Ethical approval was obtained from the Ethical Review Committee of College of Health Sciences, Mekelle University (ERC 0885/2016). Written consent was obtained from each guardian and official permission letter was written from the College of Health Sciences, Mekelle University to Ayder comprehensive specialized hospital neonatal intensive care unit (NICU). Resultswere communicated to respective physicians for beneficiary measures.

## Results

### Study participants

In the present study, a total of 317 (190 male and 127 female) neonates were participated. Age of the participants ranged 1 to 28 days and mean was 6.94 (±6.42 standard deviation (SD)). Majorities (62.1%) of the neonates were below or equal to 7 days. Weight of the neonates was between 0.95–4.3 kilograms and the mean was 2.35 (±0.73standard deviation). Majority (53.3%) of the neonates were born below 2.5 kilograms. The mean gestational period was 36.42 (±3.38) and 40.4% (128/317) were born before 37 weeks of gestation. Around 4.7% (15/317) of the neonates were born at home. Of the total, 26.3% (85/315) of the neonates were born bycesarean section. Thirty four (10.7%) neonates died of their clinical illness (Table 1).

**Table 1:** Socio-demographic characteristics of study participants (n = 317) in Ayder Comprehensive specialized hospital, Mekelle, Norththern Ethiopia, March 2017 –September 2018.

Variables	Frequency	Percent
**Age; days**		
1–7	197	62.1
8–14	81	25.6
15–21	27	8.5
22–28	12	3.8
**Sex**		
Male	190	59.9
Female	127	40.1
**Weight; Kgs**		
< 2.5	169	53.3
> = 2.5	148	46.7
**Delivery place**		
Home	15	4.7
Health facility	302	95.3
**Means of delivery**		
Cesarean section	85	26.8
Spontaneousvaginal delivery	225	71.0
Instrumental delivery	7	2.2
**Gestational age at birth; weeks**		
< 37	128	40.4
> = 37	189	59.6
**Prognosis status of the neonate**		
Improved	283	89.3
Died	34	10.7

### Neonatal sepsis

Of the total, 116 (36.6%) of the study participants were found to be with culture proven neonatal sepsis. There was no statistically significant difference in prevalence of neonatal sepsis among age groups (p > 0.05). The prevalence of neonatal septicemia was higher among females, and those who born at gestational period <37 weeks, at home and by spontaneous vaginal delivery but it was not statistically significant compared to their counter parts (AOR > 0.05), where as neonatal septicemia was higher among those whose weight was < 2.5 kgs at birth compared to those who born with > = 2.5 kgs and this was statistically significant difference (AOR = 1.64 (1.13–2.78); p = 0.04) (Table 2).

**Table 2:** Association of socio-demographic characteristics of study participants (n = 317) with culture confirmed neonatal septicemia in Ayder Comprehensive Specialized Hospital, Mekelle, Northern Ethiopia, March 2017– September 2018.

Variable	Total	Culture		COR (95%)	p-value	AOR(95%	p-value
		Positive	Negative				
**Age group; days**					0.67		
1–7	197	68(34.5)	129(65.5)	0.53(0.16–1.70)	0.28		
8–14	81	31(38.3)	50(61.7)	0.62(0.18–2.09)	0.44		
15–21	27	11(40.7)	16(59.3)	0.69(0.18–2.70)	0.59		
22–28	12	6(50.0)	6(50.0)	1			
**Sex**							
Male	190	68(35.8)	122(64.2	0.92(0.58–1.46)	0.72		
Female	127	48(37.8)	79(62.2)	1			
**Weight; Kgs**							
< 2.5	169	71(42.0)	98(58.0)	1.66(1.24–2.64)	0.03	0.04	1.64(1.13–2.78)
> = 2.5	148	45(30.4)	103(69.6	1			
**Delivery place**							
Home	15	6(40.0)	9(60.0)	1.16(0.40–3.36)	0.78		
Health facility	302	110(36.4	192(63.6	1			
**Means of delivery**					0.23		
Caesarean section	85	25(29.4)	60(70.6)	1.04(0.12–5.73)	0.96		
Spontaneous vaginal delivery	225	89(39.6)	136(60.4	1.64(0.31–8.62)	0.56		
Instrumental delivery	7	2(28.6)	5(71.4)	1			
**Gestatinal period at birth; weeks**							
<37	128	54(42.2)	74(57.8)	1.50(0.94–2.38)	0.09		
> = 37	189	62(32.8)	127(67.2	1			

### Bacterial isolates

From the total 317 specimens, 117 bacterial isolates were detected. Of the isolates, the most frequent were *Klebsiella pneumoniae* (41 isolates; 35%), *Klebsiella oxytoca* (32 isolates; 27.4%) and *Coagulase negative Staphylococcus* (11 isolates; 9.4%)respectively (Table 3). Mixed infection (*Klebsiella oxytoca* + *Acinetobacter species*) was found in one neonate.

**Table 3:** Frequency of bacterial isolates (n = 117) from septicemia suspected neonates in Ayder Comprehensive specialized hospital, Mekelle, Northern Ethiopia, March 2017–September 2018.

Species	Frequency	Percent
Coagulase negative Staphylococci species (CoNS)	11	9.4
*Staphylococcus aureus*	9	7.7
*Enterococcus* species	1	0.9
*Escherichia coli*	7	6.0
*Klebsiella pneumoniae*	41	35.0
*Klebsiella oxytoca*	32	27.4
*Klebsiella ozaenae*	3	2.6
*Klebsiella rhinoscleromatosis*	1	0.9
*Citrobacter* species	5	4.3
*Enterobacter* species	4	3.4
*Serratia* species	1	0.9
*Acinetobacter* species	2	1.7

### Associated factors

Among the assessed risk factors, hospital stayshowed statistically significant association with culture proven neonatal septicemia: neonates who stayed for greater than or equal to three days in the hospital were about 3.7 times more at risk to develop sepsis compared to those who stayed for less than three days (AOR = 3.65 (2.17–6.13), p < 0.001). Whereas factors such as blood transfusion history, intrapartum fever, HIV status of the mother, prolonged duration of labour, PROM(Premature Rupture of Membranes), prolonged PROM and PPROM did not show significant association withculture positive results (p >0.05) (Table 4).

**Table 4:** Association of possible risk factors (n = 317) with culture positive neonatal septicemia in Ayder Comprehensive specialized hospital, Mekelle, Northern Ethiopia, March 2017 –September 2018.

Variable	Total	Culture		COR (95%)	p-value	AOR(95%)	p-value
		Positive	Negative				
**Hospital stay; days**							
< 3	151	31(20.5)	120(79.5)	1		1	
> = 3	166	85(51.2)	81(48.8)	4.06(2.47–6.69)	0.00	3.65(2.17–6.13)	0.00
**Presence of Indwelling Medical device**							
Yes	177	70(39.5)	107(60.5)	1.34(0.84–2.13)	0.22		
No	140	46(32.9)	94(67.1)	1			
**Blood transfusion**							
Yes	20	8(40.0)	12(60.0)	1.17(0.46–2.94)	0.74		
No	297	108(36.4)	189(63.6)	1			
**Intrapartum fever**							
Yes	18	7(38.9)	11(61.1)	1.11(0.42–2.95)	0.84		
No	299	109(36.5)	190(63.5)	1			
**HIV status of the mother** 0.88							
Positive	6	2(33.3)	4(66.7)	0.63(0.07–5.35)	0.67		
Negative	302	110(36.4)	192(63.6)	0.72(0.19–2.7)	0.62		
Unknown	9	4(44.4)	5(55.6)	1			
**Prolonged duration of labour**							
Yes	9	2(22.2)	7(77.8)	0.49(0.10–2.38)	0.37		
No	308	114(37.0)	194(63.0)	1			
**PROM**							
Yes	28	9(32.1)	19(67.9)	0.81(0.35–1.85)	0.61		
No	289	107(37.0)	182(63.0)	1			
**Prolonged PROM**							
Yes	22	9(40.9)	13(59.1)	1.22(0.50–2.94)	0.66		
No	295	107(36.3)	188(63.7)	1			
**PPROM**							
Yes	20	9(45.0)	11(55.0)	1.45(0.58–3.62)	0.42		
No	297	107(36.0)	190(64.0)	1			

PROM = Premature Rupture of Membranes; PPROM = Preterm Premature Rupture of Membranes

With regard to the clinical presentations; jaundice, apnea and seizure showed statistically significant association with culture confirmed septicemia in binary logistic regression analysis (p<0.05). However, none of the indicated clinical signs and symptomsshowed statistically significant association with culture confirmed neonatal septicemia in multi-variant logistic regression analysis (p > 0.05). Culture confirmed septicemia did not show also significant association with the final prognosis of the neonate (Table 5).

**Table 5:** Association of clinical presentations and outcome of the study participants (n = 317) with culture positive neonatal septicemia in Ayder Comprehensive specialized hospital, Mekelle, Northern Ethiopia, March 2017 –September 2018.

Variable	Total	Culture		COR	p-value	AOR	p-value
		Positive	Negative				
**Lethargy**							
Yes	100	40(40.0)	60(60.0)	1.24(0.76–2.01)	0.39		
No	217	76(35.0)	141(65.0)	1			
**Fever**							
Yes	86	29(33.7)	57(66.3)	0.84(0.50–1.42)	0.52		
No	231	87(37.7)	144(62.3)				
**Failure to suck**							
Yes	240	89(37.1)	151(62.9)	1.09(0.64–1.87)	0.75		
No	77	27(35.1)	50(64.9)	1			
**Moro reflex**							
Yes	182	62(34.1)	120(65.9)	0.78(0.49–1.23)	0.28		
No	135	54(40.0)	81(60.0)	1			
**Tachypnea**							
Yes	172	68(39.5)	104(60.5)	1.32(0.83–2.10)	0.24		
No	145	48(33.1)	97(66.9)	1			
**Tachycardia**							
Yes	76	25(32.9)	51(67.1)	0.81(0.47–1.39)	0.44		
No	241	91(37.8)	150(62.2)	1			
**Apnea**							
Yes	42	22(52.4)	20(47.6)	2.12(1.10–4.08)	0.03	1.26(0.61–2.62)	0.53
No	275	94(34.2)	181(65.8)	1		1	
**Respiratory distress**							
Yes	68	27(39.7)	41(60.3)	1.18(0.68–2.05)	0.55		
No	249	89(35.7)	160(64.3)	1			
**Seizures**							
Yes	30	16(53.3)	14(46.7)	2.14(1.00–4.56)	0.05	1.89(0.83–4.31)	0.13
No	287	100(34.8)	187(65.2)	1		1	
**Abdominal distention**							
Yes	33	14(42.4)	19(57.6)	1.32(0.63–2.73)	0.46		
No	284	102(35.9)	182(64.1)	1			
**Meconium**							
Yes	22	5(22.7)	17(77.3)	0.49(0.18–1.36)	0.17		
No	295	111(37.6)	184(62.4)	1			
**Jaundice**							
Yes	60	30(50.0)	30(50.0)	1.99(1.13–351)	0.02	1.21(0.63–2.27)	
No	257	86(33.5)	171(66.5)	1		1	
**Delayed cry**							
Yes	69	28(40.6)	41(59.4)	1.24(0.72–2.14)	0.44		
No	248	88(35.5)	160(64.5)	1			
**NEC**							
Yes	11	6(54.5)	5(45.5)	2.14(0.64–7.17)	0.22		
No	306	110(35.9)	196(64.1)	1			
**Asphyxia**							
Yes	48	16(33.3)	32(66.7)	0.85(0.44–1.62)	0.61		
No	269	100(37.2)	169(62.8)	1			
**Prognosis**							
Improved	283	104(36.7)	179(63.3)	1			
Died	34	12(35.3)	22(64.7)	1.07 (0.51–2.24)	0.87		

NEC = Necrotizing colitis

### Antimicrobial susceptibility pattern

In this study, most isolates were resistant to the commonly used antimicrobial drugs: none of the isolates of *S*. *aureus* was susceptible to erythromycin, oxacilline, ampicillin and gentamycin. *S*. *aureus* were 66.7% (6/9) resistant to vancomycin, clindamycin and tetracycline; 55.6% (5/9) to ceftriaxone, ceftazidime, cefoxitin and co-trimoxazole; 44.4% (4/9) to chloramphenicol and ciprofloxacin. The most frequently isolated bacteria, *K*. *pneumoniae*, were found to be highly resistant with the following rate: 100% (41/41isolates) to ampicillin and amoxacillin-clavulunic acid; 90.3% (37/41) to gentamycin, ceftriaxone and ceftazidime; 82.9% (34/41) to co-trimoxazole; 63.4% (26/41) totetracycline. None of the isolates of *E*. *coli* was susceptible to ampicillin, gentamycin, tetracycline and amoxacillin-clavulunic acid. But, allisolates of *E*. *coli* (7/7) were susceptible to chloramphenicol. All isolates of Citrobacter and Enterobacter species were resistant to co-trimoxazole, gentamycin and cefotaxime (Table 6).

**Table 6:** Drug Susceptibility profiles of bacterial isolates from culture positive septicemic neonates (n = 116) in Ayder Comprehensive specialized hospital, Mekelle, Northern Ethiopia, March 2017 –September 2018.

Bacterial isolates		Antibiotics																	
		GN	TTC	CLN	CIP	CAF	AMP	CRO	AMC	E	CX	CFZ	CFO	SXT	VAN	OX	NR	AK	MRO
**CoNS (11)**	**R**	7(63.6)	9(81.2)	3(27.3)	6(54.5)	3(27.3)	8(72.7)	7(63.6)	9(81.8)	8(72.7)	7(63.6)	10(90.9		7(63.6)	8(72.7)	10(90.9			
	**I**	2(18.2)	2(18.2)	1(9.1)	2(18.2)	0(0)	3(27.3)	2(18.2)	2(18.2)	2(18.2)	2(18.2)	1(9.1)	NT	1(9.1)	1(9.1)	1(9.1)	NT	NT	NT
	**S**	2(18.2)	0(0)	7(63.6)	3(27.3)	8(72.7	0(0)	2(18.2)	0(0)	1(9.1)	2(18.2)	0(0)		3(27.3)	2(18.2)	0(0)			
***S*. *aureus* (9)**	**R**	9(100)	6(66.7)	6(66.7)	4(44.4)	4(44.4)	9(100)	5(55.6)	2(22.2)	7(77.8)	5(55.6)	5(55.6)		5(55.6)	6(66.7)	9(100)			
	**I**	0(0)	2(22.2)	1(11.1)	3(33.3)	2(22.2)	0(0)	3(33.3)	1(11.1)	2(22.2)	0(0)	3(33.3)	NT	1(11.1)	2(22.2)	0(0)	NT	NT	NT
	**S**	0(0)	1(11.1)	2(22.2)	2(22.2)	3(33.3)	0(0)	1(11.1)	6(66.7)	0(0)	4(44.4)	1(11.1)		3(33.3)	1(11.1)	0(0)			
***Enterococcusspp*(1)**	**R**	1(100)					0(0)							1(100)	0(0)	1(100)			
	**I**	0(0)	NT	NT	NT	NT	0(0)	NT	NT	NT	NT	NT	NT	0(0)	1(100)	0(0)	NT	NT	NT
	**S**	0(0)					1(100)							0(0)	0(0)	0(0)			
***E*. *coli* (7)**	**R**	5(71.4)	5(71.4)		2(28.6)	0(0)	7(100)	4(57.1)	7(100)			4(57.1)	5(71.4)	4(57.1)			0(0)	1(14.3)	2(28.6)
	**I**	2(28.6)	2(28.6)	NT	1(14.3)	0(0)	0(0)	1(14.3)	0(0)	NT	NT	2(28.6)	0(0)	0(0)	NT	NT	3(42.9)	1(14.3)	0(0)
	**S**	0(0)	0(0)		4(57.1)	7(100)	0(0)	2(28.6)	0(0)			1(14.3)	2(28.6)	3(42.9)			4(57.1)	5(71.4)	5(71.4)
***K*. *pneumoniae* (41)**	**R**	37(90.3)	26(63.4)		5(12.2)	20(48.8	41(100)	37(90.3	41(100			37(90.3	39(95.1	34(82.9			6(14.6)	5(12.2)	4(9.8)
	**I**	2(4.9)	4(9.8)	NT	13(31.7	0(0)	0(0)	1(2.4	0(0)	NT	NT	1(2.4	2(4.9)	1(2.4)	NT	NT	17(41.5	8(19.5)	5(12.2)
	**S**	2(4.9)	11(26.8)		23(56.1	21(51.2	0(0)	3(7.3)	0(0)			3(7.3)	0(0)	6(14.6)			18(43.9	28(68.3)	32(78.0)
***K*. *oxtytoca* (32)**	**R**	20(62.50	26(81.3)		6(18.8)	6(18.8)	32(100)	24(75.0	29(90.6			30(93.8	30(93.8	32(100			4(12.5)	6(18.8)	4(12.5)
	**I**	8(25.0)	2(6.2)	NT	20(62.5	0(0)	0(0)	3(9.4)	3(9.4)	NT	NT	2(6.2)	0(0)	0(0)	NT	NT	11(34.4	10(31.3)	1(3.1)
	**S**	4(12.5)	4(12.5)		6(18.8)	26(81.2	0(0)	5(15.6)	0(0)			0(0)	2(6.2)	0(0)			17(53.1	16(50.0)	27(84.4)
***K*. *ozanae* (3)**	**R**	3(100)	1(33.3)		0(0)	0(0)	3(100)	2(66.7)	3(100)			3(100)	3(100)	1(33.3)			0(0)	0(0)	0(0)
	**I**	0(0)	1(33.3)	NT	1(33.3)	0(0)	0(0)	1(33.3)	0(0)	NT	NT	0(0)	0(0)	0(0)	NT	NT	2(66.7)	1(33.3)	1(33.3)
	**S**	0(0)	1(33.3)		2(66.7)	3(100)	0(0)	0(0)	0(0)			0(0)	0(0)	2(66.7)			1(33.3)	2(66.7)	2(66.7)
***K*. *rhinoscleromatosis*(1)**	**R**	1(100)	0(0)		0(0)	0(0)	1(100)	1(100)	1(100)			1(100)	1(100)	0(0)			0(0)	0(00	0(0)
	**I**	0(0)	0(0)	NT	0(0)	0(0)	0(0)	0(0)	0(0)	NT	NT	0(0)	0(0)	1(100)	NT	NT	0(0)	0(0)	1(100)
	**S**	0(0)	1(100)		1(100)	1(100)	0(0)	0(0)	0(0)			0(0)	0(0)	0(0)			1(100)	1(100)	0(0)
***Citrobacter spp*(5)**	**R**	5(100)	2(40.0)		1(20.0)	5(100)	5(100)	4(80.0)	5(100)			4(80.0)	5(100)	5(100)			0(0)	1(20.0)	1(20.0)
	**I**	0(0)	1(20.0)	NT	1(20.0)	0(0)	0(0)	1(20.0)	0(0)	NT	NT	1(20.0	0(0)	0(0)	NT	NT	2(40.0)	0(0)	3(60.0)
	**S**	0(0)	2(40.0)		3(60.0)	0(0)	0(0)	0(0)	0(0)			0(0)	0(0)	0(0)			3(60.0)	4(80.0)	1(20.0)
***Enterobacter spp* (4)**	**R**	4(100)	3(75.0)		1(25.0)	2(50.0)	3(75.0)	3(75.0)	3(75.0)			4(100)	4(100)	4(100)			1(0)	1(25.0)	0(0)
	**I**	0(0)	1(25.0)	NT	1(25.0)	0(0)	0(0)	0(0)	0(0)	NT	NT	0(0)	0(0)	0(0)	NT	NT	1(0)	0(0)	3(75.0)
	**S**	0(0)	0(0)		2(50.0)	2(50.0)	1(25.0)	1(25.0)	1(25.0)			0(0)	0(0)	0(0)			2(50.0)	3(75.0)	1(25.0)
***Acinetobacterspp* (2)**	**R**		1(50.0)		1(50.0)	2(100)	2(100)	2(100)	2(100)			2(100)	2(100)	2(100)			0(0)	0(0)	0(0)
	**I**	NT	1(50.0)	NT	0(0)	0(0)	0(100)	0(0)	0(0)	NT	NT	0(0)	0(0)	0(0)	NT	NT	0(0)	0(0)	1(50.0)
	**S**		0(0)		1(50.0)	0(0)	0(0)	0(0)	0(0)			0(0)	0(0)	0(0)			2(100)	2(100)	1(50.0)
***Serratia spp*(1)**	**R**	1(100)	1(100)		0(0)	0(0)	1(100)	1(100)	1(100)			1(100)	1(100)				0(0)	0(0)	0(0)
	**I**	0(0)	0(0)	NT	1(100)	0(0)	0(0)	0(0)	0(0)	NT	NT	0(0)	0(0)	0(0)	NT	NT	1(100)	0(0)	1(100)
	**S**	0(0)	0(0)		0(0)	1(100)	0(0)	0(0)	0(0			0(0)	0(0)	1(100)			0(0)	1(100)	0(0)

GN: Gentamycin, TTC: Tetracyclin, CLN: Clindamycine, SXT: Sulphamethaxazole/trimethoprim, CAF: Chloramphenicole, OX:Oxaciline, VAN:Vancomycine, CIP: Ciprofloxacin, E: Erytromyacin, CX: Cefoxitin, AMC: Amoxacillin-clavulunic acid, AMP: Ampicillin, CRO: Ceftriaxone, CFZ: ceftazidime, AK: amikacin, MRO: meropenem, CFO: cefotaxime, NR: Norfloxacilin, R: Resistance, S: Susceptible, I, Intermediate, NT: Not Tested,CoNS: Coagulase negative Staphylococcus, spp: species.

## Discussion

Neonatal sepsis remains a life threatening problem in intensive care unit especially in developing countries like Ethiopia. As the epidemiology and spectrum of the causative organismsof neonatal sepsis varies over time and from place to place, continuous assessment and monitoring is in need at a local level. The present study was aimed to determine the prevalence of neonatal sepsis, bacteriological profile and drug resistance pattern at the Intensive Care Unit, Ayder Comprehensive Specialized Hospital, Tigray, north Ethiopia.

In our study, a high prevalence (36.6%) ofsepsis was found at theneonatal intensive care unit, Ayder Comprehensive Specialized Hospital, Mekelle, Tigray, North Ethiopia. Similar findings were reported from Gondar Ethiopia 32.1% [12], Tanzania 39% [20], Egypt 40.7% [16] and India 41.6% [18]. However, the present finding was lower compared to findings from Sudan (67.5%) [21], Chennai India 60.4% [22], Pakistan 62.8% [23], Gondar Ethiopia 46.6% [11], and Addis Ababa Ethiopia 44.7% [14]. Thepresent finding was higher compared to the reports from Nepal 15.1% [24], Nigeria 22% [25], Tanzania 24% [17] andAddis Ababa Ethiopia 27.9% [13]. The possible explanation for the difference in prevalence may be due over time change and variations from region to region.

In the present study, majority of the isolates were *Klebsiella* species. Similarly, *Klebsiella pneumoniae* was the most frequently isolated organism from other previous studies: India [15, 18, 22, 26] and Addis Ababa Ethiopia [14]. Though not a predominant, *Klebsiella* species were also among the most common pathogens reported in bloodstream infections from other previous findings in Ethiopia (13), and Egypt [16]. *Klebsiella*species arenow becoming important public health concern especially in health care settingswith only few antibiotics to treat [27, 28]. The high prevalence of *Klebsiella* species in the present study could be related to the ability of the organism to spread from patient to patient via contaminated hands of healthcare personnel or other persons, by contamination of the environment, or when the patients are on medical tools such as breathing machines and intravenous catheters. As *Klebsiella* species are ubiquitous in nature which can be found in the environment and on the mucosal surface of humans, the organism can be restrained in the hospital settings which enable to cause different infections among sick patients when there is lack of immunity, environmental and personal hygiene [28].

Presence of significant isolates of *Citrobacter*, *Enterobacter*, *Acinetobacter and Serratia species* was the other interesting finding of our study. As the infections caused due to these organisms are mostly nosocomial, the source of infection could be from the use of medical devices such as venous catheters, mechanical ventilators, or endogenously when the normal flora state alters due weakened immunity.

Of the assessed variables, length of hospital stay and the weight of the neonate showed significant association with presence of septicemia in the present study. Neonates who stayed greater as inpatient in the hospital were at high risk to develop bacterial septicemia and this may be related with the high probability for continuous exposure and acquisition of the causative bacteria from the hospital setting. Neonates who had low birth weight were at high risk to develop bacterial septicemia and the possible reason might be low birth weighted neonates are more likely to be with immature immunity and may have other co-morbidity that may easily expose them for secondary infections. The other possible reason could be low birth weighted neonates are mostly helped with different medical devicesin health care’s and this might increase the risk of exposure to numerous infections. However, other socio-demographic and clinical related variables did not show significant association with blood culture proven bacterial septicemia.

Nowadays drug resistance is becoming a major global challenge, and particularly in intensive care unit the rate is overwhelming because of the vulnerability of the patients due to reduced immunity, excessive use of broad spectrum antibiotics, invasive medical devices and procedures, and prolonged hospital stays. In the present study, most of the isolates were with frightening result to the commonly used antimicrobial drugs.

*K*. *pneumoniae*, the most frequently isolated bacteria, was 90% to100% resistant to the common antibiotics: ceftazidime, ceftriaxone, gentamycin, amoxacillin-clavulunic acid and ampicillin. Similarly, resistance rate of *E*. *coli*, and *Enterobacter* and *Citrobacter* species were 57% to100%, 75% to 100% and 80% to 100% respectively to the above mentioned antibiotics. The high resistance rate of these isolates could be attributed to the wide prescription habitof broad spectrum antibiotics in the intensive care unit and in the hospital as a general. In the present study, all isolates of *S*. *aureus* were resistant to oxacilline, ampicillin and erythromycin and gentamycin, and furthermore 55.6% *S*. *aureus* isolates were MRSA using cefoxitine as screening method.

WHO recommends ampicillin plusgentamicin for empiric treatment of neonates with suspected clinical sepsis [29]. Our study however, showed that these antibiotics are highly resisted by most of the isolated bacteria and this could be due to wide and over prescription of these antibiotics. On the other hand, since the antibiotics like meropenem and amikacin showed better antibiotic activityagainst the isolated bacterial species, under regular monitoring,these are recommended to be used in the hospital for the treatment of neonatal septicemia. In general, the high prevalence and multi-drug resistant bacteria highlights the need for strengthening of infection prevention and control strategy as well as regular monitoring of antimicrobial resistance in the hospital and especially at the intensive care unit. The present studyalso highlights a specialdue attention to be given for *Klebsiella* species (particularly *Klebsiella pneumoniae* and *Klebsiella oxytoca*) as the frequency of these organisms is found significantly high. Undergoing research with large sample size including in other hospitals will help see the bacterial distribution and drug resistanceprofile in the region, and then to develop local treatment guide for neonatal septicemia. Finally we recommended strengthening of hospital infection control and program to combat nosocomial infection such as the *Klebsiella* species dominantly isolated in this study.
